# ‘Felt sense of anomaly’‐type transdiagnostic dissociative experiences in adolescents: Endorsed phenomenology and plausible mechanisms

**DOI:** 10.1002/jcv2.70116

**Published:** 2026-04-04

**Authors:** Emma Černis, Milan Antonović, Katie Lofthouse, Lottie Shipp, Polly Waite

**Affiliations:** ^1^ School of Psychology University of Birmingham Birmingham UK; ^2^ Institute for Mental Health University of Birmingham Birmingham UK; ^3^ Norwich Medical School University of East Anglia Norwich UK; ^4^ Department of Experimental Psychology University of Oxford Life and Mind Building Oxford UK

**Keywords:** adolescence, depersonalisation, dissociation, felt sense of anomaly, measurement, psychometrics, young people

## Abstract

**Background:**

Research indicates dissociative experiences (DE) are prevalent in adolescents. However, the exact phenomenology and underlying mechanisms of dissociation in adolescence have yet to be clarified. The current study explores the presentation of, and possible psychological factors maintaining, dissociation in this population.

**Methods:**

Two online self‐report surveys captured data from 3076 adolescents aged 13–18 years. Participants completed the Černis Felt Sense of Anomaly (ČEFSA) scale of felt sense of anomaly‐type dissociation, alongside measures of mechanisms chosen to test the relevance of a cognitive‐behavioural model of dissociation in adults (cognitive appraisals, alexithymia, healthy affect regulation, and affect intolerance in the form of expressive suppression (ES)). *N* = 409 completed the ČEFSA scale at a second timepoint, one month later, enabling exploration of proposed maintenance factors.

**Results:**

Most (91.87%) adolescents endorsed at least one ČEFSA item. The mean number of endorsed items was 13.00 (SD = 9.67). The most endorsed factors of the ČEFSA were Altered Sense of Agency (82.96%), Anomalous Experience of the Self (76.76%), and Altered Sense of Connection (73.76%). Mediation analysis indicated that affect intolerance (ES) mediated the relationship between Time One and Time Two dissociation scores: greater suppression was associated with greater dissociation 1 month later. No other tested variables showed statistically significant mediation.

**Conclusion:**

Adolescents are likely to experience dissociation as detachment and disconnection, particularly relating to their selfhood and external world (i.e., depersonalisation and derealisation). This study suggests that the key element of a recent cognitive‐behavioural model of DE in adults—that affect intolerance perpetuates dissociation—may also be applicable in adolescence.

## Key Points

**What's known?**

- Dissociative experiences (DE) are prevalent in adolescence, but their phenomenology has not been characterised.
- A recent theoretical model proposes affect intolerance maintains transdiagnostic dissociation, but this has yet to be tested in adolescence.

**What's new?**

- 91.87% of the 3076 adolescents surveyed reported experiencing at least one dissociative experience ‘often’ or ‘always’ over the past fortnight.
- Only 8.13% of participants did not rate any of the 35 items as occurring ‘often’ or ‘always’.
- The ‘expressive suppression’ (ES) emotion regulation strategy was a significant mediator of dissociation scores over 1 month.

**What's relevant?**

- DE are common in adolescence—particularly derealisation and sensations of feeling ‘automatic’.
- Suppression or avoidance of emotions as a coping or regulation strategy may maintain dissociation.

## INTRODUCTION

Dissociative experiences (DE) appear to be important in adolescence. Describing highly subjective experiences of profound disconnection, emotional numbness, unreality or ‘strangeness’, evidence suggests that under the age of 18, dissociation is robustly associated with deliberate self‐harm and suicide risk (Tanaka et al., 2023; Vine et al., 2020; Černis et al., 2019), and with alcohol and drug abuse (Tolmunen et al., 2007).

Adolescence is a time of life with an increased risk of psychopathology (Powers & Casey, 2015), when many mental health problems first emerge (Sadler et al., 2018; Solmi et al., 2022). Previous work demonstrates links between dissociation and mental health difficulties common in childhood and adolescence; for example, heightened symptoms of anxiety (Lofthouse et al., 2023), panic disorder (Shipp et al., 2023), and even psychosis (Cole et al., 2016). Further, recent studies in adults implicate dissociation as a possible causal factor in symptoms of serious mental illness (Černis, Evans, et al., 2021; Černis, Molodynski, et al., 2022), indicating that understanding dissociation in adolescence could also be valuable for prevention and early intervention.

Thus, the aim of the present study is to explore the phenomenological presentation of ‘felt sense of anomaly’‐type DE in adolescents, and begin to identify their plausible psychological mechanisms in order to inform future treatment development.

### Dissociation in adolescence

DE encompass a wide range of phenomena, including detachment from oneself (including in the experience of one's own cognitions, memory, and body: i.e., depersonalisation); identity alteration (feeling unfamiliar to oneself, or seeming very different between contexts), and disconnection from the surrounding world (derealisation) (Steinberg, 1994). People suffering with these highly subjective experiences typically find them difficult to put into words, but may describe themselves as ‘living in a bubble’ or ‘behind a pane of glass’ (Černis et al., 2020).

In clinical adolescent populations, such experiences are prevalent. In a study of almost one hundred French‐speaking inpatients in Belgium, aged 12–20 years, Goffinet and Beine (2018) found that between 33% and 65% showed ‘pathological dissociative symptoms’, and 45% scored within the range of a dissociative disorder on the Adolescent Multidimensional Inventory of Dissociation. Rates of dissociation in outpatients may be lower: Vine et al. (2020) carried out ecological momentary assessment (EMA) over four days with a group of 162 young people aged 11–13 years referred to paediatric primary care and outpatient mental health clinics. They found 17%–35% of the group endorsed questions about dissociation. However, these were limited to five questions, two of which assessed feeling ‘empty’ and ‘bored’, which may arguably capture feelings unrelated to any DE. While these studies give a clear signal that dissociation is present in this age group, it is important to recognise the limitations of self‐report measures in capturing this phenomena (Fusar‐Poli et al., 2016).

Higher rates of DE may be expected in clinical groups, where levels of distress are also higher. However, relatively high rates of DE are also found in non‐clinical groups. For example, clinical levels of depersonalisation—another subtype of dissociative experience—have been found at a rate of 11.9% in a representative survey of 3809 pupils in Germany aged 12–18 years (Michal et al., 2015); and 11% of 198 psychology undergraduates at a USA university (mean age 19.72 years; Myers & Llera, 2020). A recent meta‐analysis of 76 studies of DE and dissociative disorders in college students indicated that 17% of students self‐report having DE, and 11.4% met criteria for a dissociative disorder (Kate et al., 2020). In addition, there is preliminary evidence that the frequency of DE may be higher in adolescents and young adults than in later adulthood (Ross et al., 1989; Černis et al., 2023).

However, existing literature has yet to determine whether this replicated pattern reflects levels of DE globally, or whether it is driven by specific experiences of DE. That is to say, the phenomenology of the high dissociation in adolescence has yet to be characterised. Further, taking a transdiagnostic approach to DE may be particularly valuable in this group, where the incidence of new mental health difficulties is at its peak (Uhlhaas et al., 2023), but where emphasis on diagnosis may be ‘unable to capture the complex, nonspecific, and dynamic emerging phases of mental disorders’ (Díaz‐Caneja & Guloksuz, 2024).Černis and colleagues have outlined a transdiagnostic subset of DE experienced as a subjective ‘felt sense’ of strangeness (Černis, Beierl, et al., 2021; Černis, Molodynski, et al., 2022). Subjectively ‘strange’ sensations include unfamiliarity, unreality, automaticity, or disconnection which may affect one or more ‘domains’ of experience, such as perception, identity, cognition, or the physical body (Černis, Beierl, et al., 2021; Černis et al., 2020). This construct is conceptualised as a subtype of dissociation, in response to a need for greater clarity and precision when referring to DE, and is thus referred to as ‘felt sense of anomaly’‐type dissociation (FSA‐dissociation).

As outlined by Černis, Beierl, et al. (2021), FSA‐dissociation has phenomenological overlaps with the constructs of depersonalisation and derealisation. It should be noted, however, that Lofthouse et al. (2023) found that state anxiety in adolescents predicted FSA‐dissociation—but not depersonalisation—suggesting that these constructs may have different underlying mechanisms, despite phenomenological similarities.

FSA‐dissociation also demonstrates significant phenomenological overlap with anomalous self‐experiences in the psychosis literature (e.g., Parnas & Handest, 2003). However, the same distinctions noted between depersonalisation and anomalous self experiences are likely to be true also for FSA‐dissociation. In particular, it has been noted that ASEs involve disturbances in cognition and self/other boundaries, which are not present in depersonalisation (Sass et al., 2013).

While important, discrimination between FSA‐dissociation, depersonalisation, and anomalous self‐experiences is not addressed in the current study, which focuses on phenomenology and psychological mechanisms within a single, theoretically defined dissociation subtype (FSA‐dissociation).

### Mechanisms of FSA‐dissociation in adolescence

The second aim of this study is to explore whether the mechanisms implicated in the maintenance of FSA‐dissociation in adults (Černis, Ehlers, & Freeman, 2022; Černis et al., 2025) might also be relevant to transdiagnostic DE in adolescence. This is an important question, since such mechanisms are used as the basis of treatment development work (Clark, 2004).

**FIGURE 1:**
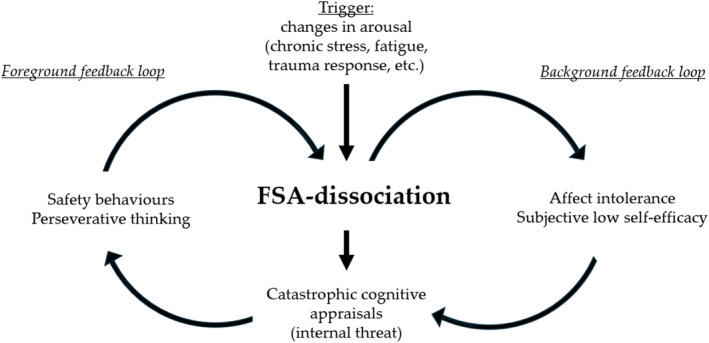
Theoretical cognitive‐behavioural model of felt sense of anomaly‐type dissociation (Černis et al., 2025).

Černis and colleagues suggest a cognitive‐behavioural model of FSA‐dissociation, informed by clinical practice and lived experience input, whereby changes in internal states (arousal) are interpreted as an ‘internal threat’ (Černis, Ehlers, & Freeman, 2022; Černis et al., 2025). In this theoretical model (Figure 1), FSA‐dissociation is maintained by a classical cognitive‐behavioural (‘foreground’) feedback loop: catastrophic cognitive appraisals of the DE, rumination, and safety behaviours inadvertently reinforce the DE. The second half of the model explains why DE are interpreted catastrophically: a second, complementary (‘background’) feedback loop of affect intolerance (i.e., aversion to any embodied emotional response, positive or negative) and subjective low self‐efficacy provide a context for interpreting changes in arousal as a form of internal threat and thus also reinforce DE. Černis, Johns, et al. (2025) suggest that alexithymia may also be important in understanding FSA‐dissociation, as individuals who struggle to make sense of the changes in their internal state may be more prone to affect intolerance.

The application of this hypothesised model to adolescents has already found some support in recent cross‐sectional studies. Lofthouse et al. (2023) demonstrated a role for rumination and catastrophic cognitive appraisals in the relationship between FSA‐dissociation and trait anxiety, while Shipp et al. (2023) found significant correlations between FSA‐dissociation, emotion regulation strategies (encompassing an adaptive and maladaptive (avoidant) strategy), alexithymia, and catastrophic cognitive appraisals.

However, while both of these previous studies demonstrate correlation between FSA‐dissociation and its proposed maintenance mechanisms in adolescence, neither has explored whether FSA‐dissociation is mediated by these proposed mechanisms.

### Study aims and hypotheses

The first aim of the current study is to better understand the phenomenology of adolescent transdiagnostic DE by evaluating endorsement rates for items and factors of the Černis Felt Sense of Anomaly (ČEFSA) scale (Černis, Beierl, et al., 2021)—a measure of the FSA‐dissociation construct. This specific subtype has been selected because it represents a theoretically coherent and clinically salient subtype of dissociative experience that has been proposed in recent literature, but has not yet been fully examined in adolescence.

The second aim is to assess whether the mechanisms proposed to maintain FSA‐dissociation in adults mediate the relationship between adolescents' FSA‐dissociation scores over a 1‐month timeframe. Two factors drawn directly from Černis, Johns, et al.'s, (2025) theoretical model (catastrophic cognitive appraisals and affect intolerance) will be tested.

Here, affect intolerance is operationalised as use of the maladaptive emotion regulation strategy ‘ES’, which involves avoidance and denial of affect. It is hypothesised that both ES and catastrophic cognitive appraisals will mediate FSA‐dissociation scores, in line with the theoretical model.

Two further factors (not drawn directly from the model) will also be tested as mediators of FSA‐dissociation scores in adolescents: alexithymia and a healthy emotion regulation strategy (cognitive reappraisal).

Alexithymia has been suggested as a possible cause of affect intolerance in the context of FSA‐dissociation (Černis et al., 2025), and its inclusion in the theoretical model of FSA‐dissociation has been considered throughout the model's development (Černis, Ehlers, & Freeman, 2022; Černis, Molodynski, et al., 2022). It is therefore important to test whether alexithymia confers any additional effect, independent from affect intolerance, in this group. Thus, alexithymia is hypothesised to have a positive relationship with FSA‐dissociation scores, but it is hypothesised not to have a significant mediation effect, in‐line with its omission from the theoretical model.

Finally, cognitive reappraisal describes an adaptive method for regulating affect that involves reinterpreting the emotional experience. It could therefore arguably be seen as the inverse of catastrophic cognitive appraisals and affect intolerance, and would therefore be anticipated to have a negative relationship with FSA‐dissociation. However, this makes it unclear whether cognitive reappraisal should be expected to have a significant (negative) mediation effect.

## METHOD

### Design & procedure

To increase sample size and assess a broad range of responses, data from two online self‐report questionnaire studies were combined. Both received approval from the University of Oxford Medical Sciences Interdivisional Research Ethics Committee (R71497/RE001 and R77368/RE001). Both previous studies were pre‐registered (https://osf.io/hzp8x/ and https://osf.io/jd973/). The current study was not pre‐registered.

These studies are described fully elsewhere (Lofthouse et al., 2023; Shipp et al., 2023), but can be summarised as follows: both recruited via social media (Lofthouse et al. (2023) additionally recruited via UK schools), to collect data using Qualtrics (Qualtrics, 2020, 2021), including collection of informed consent and assent according to British Psychological Society (2021) guidance. Respondents aged 16–18 years were able to provide informed consent following presentation with the participant information sheet: parental consent was not required. Respondents aged 13–15 years were requested to pass the device to a parent or guardian, who was presented with a parent information sheet, and the option to provide written informed consent for their child to take part. Afterwards, the adolescent was presented with an information sheet and provided written assent.

Following the consent/assent process, participants proceeded to the self‐report measures. The final page of the survey contained debrief material including relevant self‐help resources.

Additionally, Shipp et al. (2023) collected longitudinal data. One month after the first survey, participants were emailed a link to complete the same measures a second time.

### Participants

To be eligible, participants had to be aged 13–18 years, based in the UK, and have a sufficient level of English to engage with the research materials. The inclusion of under‐represented groups was considered in the selection of schools approached in the (Lofthouse et al., 2023) study.

Participant datasets for Lofthouse et al. (2023) and Time 1 of Shipp et al. (2023) were combined, resulting in a total number of 3076 participants for the Aim 1 analysis (see below). The 409 participants who provided longitudinal data in (Shipp et al., 2023) were included in the analysis for Aim 2. Table 1 summarises the characteristics of these groups.

**TABLE 1:** Demographic data and descriptive statistics for participant groups.

Participant group	Aim 1	Aim 2
*N*	3076	409
Age		
Mean (SD)	16.23 (1.45)	16.72 (1.36)
Gender *N* (%)		
Female	2049 (66.61%)	284 (69.4%)
Male	533 (17.33%)	53 (13.0%)
Other	403 (13.10%)	61 (14.9%)
Prefer not to say or missing	91 (2.96%)	11 (2.7%)
Ethnicity *N* (%)		
White (any)	2605 (84.69%)	356 (87.0%)
Mixed/Multiple	184 (5.98%)	19 (4.6%)
Asian (any)	172 (5.59%)	22 (5.4%)
Black (any)	56 (1.82%)	3 (0.7%)
Other	33 (1.07%)	8 (2.0%)
Prefer not to say or missing	26 (0.84%)	1 (0.2%)
Measure mean (SD)		
CAD‐P	26.85 (12.31)	T1 26.60 (11.94); T2 26.03 (12.71)
ČEFSA	67.04 (31.39)	T1 64.99 (31.38); T2 64.47 (33.45)

Abbreviations: CAD‐P, Cognitive Appraisals of Dissociation in Psychosis; ČEFSA, Černis Felt Sense of Anomaly Scale; T1, Time 1 (0 months); T2, Time 2 (1 month).

### Measures

Following demographic questions (age, gender, and ethnicity), participants completed self‐report measures, the psychometrics for which can be found in Shipp et al. (2023).

#### Černis felt sense of anomaly (ČEFSA) scale (Černis, Beierl, et al., 2021)

With seven factors capturing key domains and types of FSA‐dissociation, the ČEFSA provides a comprehensive measure of transdiagnostic DE. The full version is a 35‐item scale including items such as ‘I feel like a stranger to myself’ and ‘I feel detached from my emotions’ to measure experiences of anomalous self, body, and affect, and altered subjective senses of familiarity, connection, agency, and reality. Respondents rate the frequency of each item over the last 2 weeks on a five‐point Likert‐type scale from 0 (‘never’) to 4 (‘always’). The total score of the scale is the sum of all items (range 0–140), with higher scores indicating greater frequency of DE. The scale was found to have excellent internal consistency in this group (Cronbach's alpha = 0.972), and the recent development of a short‐form of the scale indicated that the majority of items are age‐invariant (Černis et al., 2023). Cronbach's alphas for the subscales were similarly high in this group (Self = 0.817; Body = 0.906; (Un)Familiarity = 0.900; Emotion = 0.938; (Dis)Connection = 0.895; Agency = 0.821; (Un)Reality = 0.895).

#### Cognitive appraisals of dissociation in psychosis measure (CAD‐P; Černis, Bird, et al., 2021)

The Cognitive Appraisals of Dissociation in Psychosis ,CAD‐P assesses catastrophic cognitive appraisals of dissociation arising in the context of psychosis, though the items reflect appraisals that are applicable in a non‐clinical adult group (Černis, Ehlers, & Freeman, 2022). It consists of 13 items, such as ‘I can't trust my own mind’, rated on a 5‐point Likert scale (0 = ‘never’, 4 = ‘always’). The total sum score ranges between 0 and 52, with higher scores reflecting greater frequency of negative cognitive appraisals of dissociation. Cronbach's alpha in this group was 0.917.

#### The emotion regulation questionnaire for children and adolescents (ERQ‐CA; Gullone & Taffe, 2012)

The ERQ‐CA is an adaptation of the adult Emotion Regulation Questionnaire (Gross & John, 2003) comprising 10 items rated on a 5‐point Likert scale (1 = ‘strongly disagree’ and 5 = ‘strongly agree’). It has two subscales: cognitive reappraisal (e.g., ‘When I'm worried about something, I make myself think about it in a way that helps me feel better’) (score range 6–30, with higher scores reflecting greater cognitive reappraisal), and ES (e.g., ‘I keep my feelings to myself’) (score range 4–20, with higher scores reflecting greater cognitive reappraisal). Cognitive reappraisal is interpreted here as an adaptive emotion regulation strategy, which may be indicative of low affect intolerance, whereas ES can be interpreted as a behavioural marker of affect intolerance. In this group, Cronbach's alphas were 0.729 for cognitive reappraisal, and 0.753 for ES.

#### Alexithymia questionnaire for children (AQC; Rieffe et al., 2006)

Two subscales of the AQC (Difficulty Identifying Feelings and Difficulty Describing Feelings) were used, totalling 12 items. Each item is rated on a scale from 0 to 2 (0 = ‘not true’, 2 = ‘true’), giving a score range of 0–24, with higher scores reflecting greater cognitive reappraisal. This version of the scale has been tested previously in an adolescent group and showed good psychometric properties (Cronbach's *α* = 0.83) (Loas et al., 2017) (0.772 in the current group).

### Statistical analysis

Statistical analysis was conducted using SPSS [version 29.0.0.0] (IBM Corp, 2023). To identify anomalous responses, scores were either checked with the intention to exclude data where these deviated from the mean by 1.5 times the interquartile range (Lofthouse et al., 2023; Tukey, 1977), or inspected visually for inappropriate responding (e.g., selecting exclusively minimum or maximum values) (Shipp et al., 2023). Using these methods, no anomalous data were identified. Regarding missing data, responses were only included in the current analyses where participants had completed a minimum of 80% of the relevant questionnaires. Missing responses comprised less than 1% of the data. These were treated as being missing at random and were deleted pairwise for Aim 1. For Aim 2, missing cases were deleted listwise.

Data were assessed for Normality, skewness, and kurtosis. According to Kolmogorov‐Smirnov and Shapiro‐Wilk values, the normality of the distribution of scores was significantly different from a normal distribution on all measurements. However, skewness and kurtosis analysis showed no substantial skewness or kurtosis (within ± 1.0 range) and the use of parametric testing was justified in Aim 2 due to the power provided by the large sample size (Ghasemi & Zahediasl, 2012).

#### Aim 1: Phenomenology of DE in adolescence

A ČEFSA item was considered ‘endorsed’ if it was rated as being experienced ‘often’, or ‘always’ over the past 2 weeks (scores of 3 or 4). Frequencies of endorsement were calculated per item, per scale factor, and as a proportion of the whole scale.

#### Aim 2: Mechanisms of DE in adolescence

Mediation of the relationship between ČEFSA scores at Time 1 and Time 2 in the Shipp et al. (2023) longitudinal data by (Time 1) cognitive appraisals, cognitive reappraisal, emotional suppression, and alexithymia was assessed using bootstrapped mediation analysis (Hayes, 2018). 95% confidence intervals (CI) were generated using 5000 bootstrap samples. Finally, multiple linear regression was used to determine the amount of variance explained by each mediator. All significance testing was two‐tailed.

## RESULTS

The demographic characteristics of participants for Aim 1 and Aim 2 are shown in Table 1. The majority of the Aim 1 group were female and white, with an average age of 16.23 years. The mean score for the ČEFSA scale in this group was 67.04. The Aim 2 group were representative of the larger Aim 1 sample (majority white and female, average age 16.72, mean ČEFSA score 64.99).

### Aim 1: Phenomenology of DE in adolescence

**FIGURE 2:**
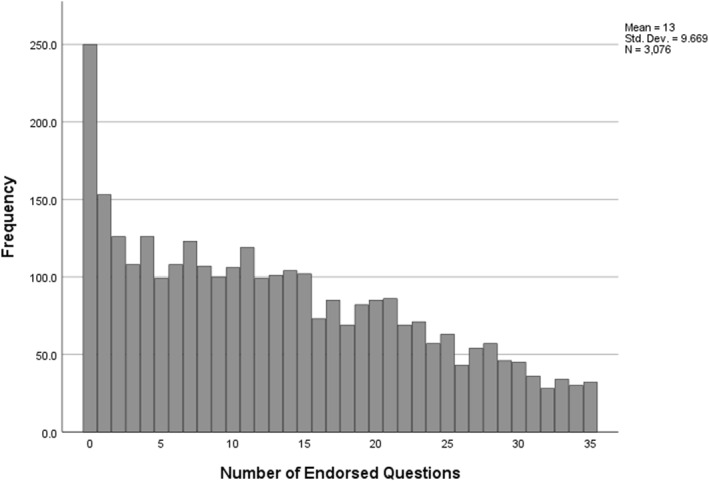
Graph showing the number of participants endorsing ČEFSA items.

On average, adolescents endorsed 13 of 35 ČEFSA items and 91.87% of participants endorsed at least one FSA‐dissociative experience as happening ‘often’ or ‘always’ over the past two weeks (Table 2; Figure 2). Only 8.13% of respondents did not endorse any items of the scale. 11.8% endorsed at least 75% of the items, 32.1% endorsed at least 50%, and 61% endorsed at least 25% of the 35 items. The most highly endorsed scale factor was Altered Sense of Agency (mean 2.43 of 5 items endorsed), and the least was Altered Sense of Familiarity (mean 0.96 out of 5 items).

**TABLE 2:** Rates of endorsement for the ČEFSA (whole scale and factors) (*n* = 3076).

	No. of items endorsed	No. participants endorsing	No. participants endorsing
	Mean (SD)	0 items *N* (%)	≥1 item *N* (%)
ČEFSA (whole scale)	13.00 (9.67)	250 (8.13)	2826 (91.87)
Scale factors			
Altered sense of agency	2.43 (1.65)	524 (17.04)	2552 (82.96)
Altered sense of connection	2.27 (1.86)	807 (26.24)	2269 (73.76)
Anomalous experience of emotion	2.12 (2.03)	1155 (37.55)	1921 (62.45)
Anomalous experience of the self	2.09 (1.63)	715 (23.24)	1619 (76.76)
Altered sense of reality	1.74 (1.79)	1213 (39.43)	1863 (60.57)
Anomalous experience of the body	1.39 (1.70)	1447 (45.04)	1629 (54.96)
Altered sense of familiarity	0.96 (1.44)	1789 (58.16)	1287 (41.84)

The three most endorsed individual items (Table 3) were ‘I find myself drifting off into my own world when I'm with others' (63.98% of respondents), ‘I don't notice how much time passes' (60.43%), and ‘I feel disconnected from other people’ (56.40%). The least endorsed item was ‘familiar sights, smells (etc.) feel unfamiliar to me’ (13.56%).

**TABLE 3:** Response and endorsement rates for each Černis felt sense of anomaly scale item as % of the participant group.

		Never	Rarely	Sometimes	Often	Always	Item endorsed (i.e., often or always)
1.	I feel like a stranger to myself. [S]	16.38	16.48	22.33	24.02	20.77	44.79
2.	I feel detached from my physical body (or parts of it). [B]	24.48	20.64	22.59	19.38	12.87	32.25
3.	Places that I know seem unfamiliar. [F]	32.90	27.89	21.75	12.71	4.68	17.39
4.	I don't fully experience emotions. [E]	13.17	15.25	23.15	26.59	21.81	48.40
5.	I feel disconnected from the world around me. [C]	10.73	11.61	23.93	32.64	21.07	53.71
6.	I'm absorbed in my own world and don't notice what is happening around me. [A]	8.29	15.34	24.84	32.38	19.12	51.50
7.	I feel like other people aren't real. [R]	21.65	16.81	24.58	21.26	15.70	36.96
8.	My personality changes seemingly at random. [S]	10.44	13.13	23.24	26.17	27.02	53.19
9.	My body (or parts of it) feels like it doesn't belong to me. [B]	32.61	20.77	21.07	14.30	11.22	25.52
10.	Familiar sights, smells (etc.) feel unfamiliar to me. [F]	37.45	27.70	21.23	9.56	4.00	13.56
11.	I can't feel emotions. [E]	20.48	20.03	26.69	22.24	10.50	32.74
12.	I feel disconnected from other people. [C]	7.70	10.37	25.49	35.27	21.13	56.40
13.	I find myself drifting off into my own world when I'm with others. [A]	5.59	8.39	21.98	35.86	28.12	63.98
14.	The world seems like it is fake. [R]	18.24	14.47	22.46	23.93	20.90	44.83
15.	I feel like I don't have a personality. [S]	16.16	13.72	22.17	24.12	23.73	47.85
16.	My body (or parts of it) feels unreal or strange. [B]	29.94	22.30	22.82	15.57	9.27	24.84
17.	People around me seem different or altered. [F]	24.06	20.25	27.02	19.34	9.20	28.54
18.	I feel detached from my emotions. [E]	14.11	13.91	25.46	26.98	19.54	46.52
19.	I feel as if I'm experiencing life from very far away. [C]	23.60	18.37	26.20	18.63	13.20	31.83
20.	I don't notice how much time passes. [A]	7.48	10.05	22.04	31.66	28.77	60.43
21.	The world around me seems unreal. [R]	20.32	16.25	25.29	22.04	16.03	38.07
22.	I act like someone else without meaning to. [S]	13.59	14.99	25.65	27.44	18.27	45.71
23.	My body feels like it's not under my control. [B]	26.04	21.75	24.97	16.84	10.31	27.15
24.	People I know seem unfamiliar. [F]	33.00	23.67	24.84	13.10	5.40	18.50
25.	I feel disconnected from my emotions. [E]	15.80	15.05	25.23	26.17	17.72	43.89
26.	The things happening around me seem unreal to me—like a dream or a movie. [C]	17.59	16.12	26.17	24.67	15.44	40.11
27.	I lose track of my surroundings. [A]	16.87	18.04	27.63	25.16	12.22	37.38
28.	I feel as though other people stop existing when I can't see them. [R]	32.83	17.30	20.25	15.34	14.24	29.58
29.	I feel like I'm more than one person. [S]	45.68	18.79	17.72	9.53	8.22	17.75
30.	My body feels numb. [B]	24.90	19.70	25.68	19.67	9.98	29.65
31.	Things I've done many times before seem new or unfamiliar. [F]	32.90	25.00	24.32	13.46	4.32	17.78
32.	My emotions don't seem real. [E]	20.06	15.51	24.22	23.89	16.29	40.18
33.	I feel detached from what I'm doing. [C]	14.04	13.17	27.47	30.62	14.66	45.28
34.	I feel like an alien or a ghost. [R]	38.65	17.56	19.44	15.44	8.84	24.28
35.	I freeze, unable to do anything. [A]	25.10	20.25	25.10	20.87	8.68	29.55

*Note*: [A] = item belongs to the altered sense of agency factor; [C] = altered sense of connection factor; [E] = anomalous experience of emotion factor; [S] = anomalous experience of the self; [R] = altered sense of reality; [B] = anomalous experience of the body; [F] = altered sense of familiarity.

### Aim 2: Mechanisms of DE in adolescence

**FIGURE 3:**
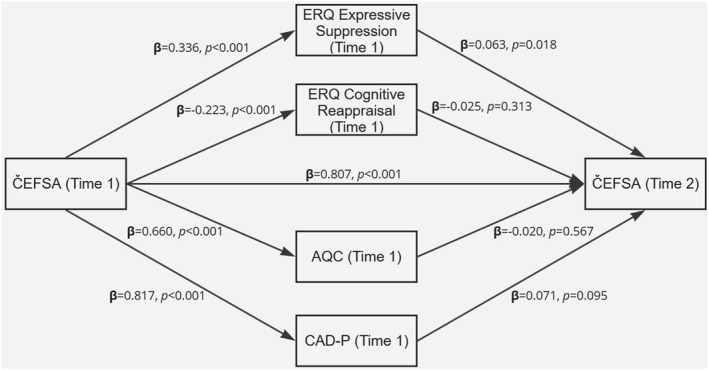
The relationship between ČEFSA scores at Time 1 and Time 2, and proposed mediating factors. All reported coefficients are standardised. Significance at *p* < 0.05.

The relationship between Time 1 and Time 2 ČEFSA scores and the potential mediators are presented in Figure 3, and the unstandardised and standardised statistics for the mediation and regression analyses are presented in Table 4.

**TABLE 4:** Statistics for the regression and mediation analyses (*n* = 409).

Regression analysis	*b*	*β*	Standard error	*t*	*p*	95% confidence interval	
						Lower	Upper
ČEFSA time 1 on ERQ expressive suppression time 1	0.0362	0.337	0.005	7.208	<0.001	0.026	0.046
ČEFSA time 1 on ERQ cognitive reappraisal time 1	−0.0285	−0.223	0.006	−4.619	<0.001	−0.041	−0.016
ČEFSA time 1 on AQC time 1	0.0921	0.660	0.005	17.743	<0.001	0.082	0.102
ČEFSA time 1 on CAD‐P time 1	0.311	0.817	0.011	28.585	<0.001	0.290	0.332
ERQ expressive suppression time 1 on ČEFSA time 2	0.626	0.0631	0.263	2.383	0.018	0.110	1.142
Cognitive reappraisal time 1 on ČEFSA time 2	−0.205	−0.0246	0.203	−1.010	0.313	−0.604	0.194
AQC time 1 on ČEFSA time 2	−0.149	−0.0196	0.261	−0.573	0.567	−0.662	0.363
CAD‐P time 1 on ČEFSA time 2	0.198	0.0708	0.118	1.674	0.095	−0.035	0.431

**Mediation analysis**	**Unstandardised effect**	**Standardised effect**	**Standard error**	***t***	***p***	**95% confidence interval**	
						**Lower**	**Upper**
Total effect of time 1 ČEFSA on time 2 ČEFSA	0.879	0.936	0.0252	37.097	<0.001	0.887	0.986
Direct effect of time 1 ČEFSA on time 2 ČEFSA	0.807	0.860	0.0455	18.898	<0.001	0.770	0.949

**Indirect effects of time 1 ČEFSA on time 2 ČEFSA**	**Unstandardised effect**		**Standardised effect**	**Bootstrapped standard error**		**Bootstrapped 95% confidence interval**	
						**Lower**	**Upper**
Via ERQ expressive suppression time 1	0.0226		0.0212	0.0105		0.0033	0.0450
Via ERQ cognitive reappraisal time 1	0.0058		0.0055	0.0061		−0.0055	0.0189
Via AQC time 1	−0.0138		−0.0129	0.0220		−0.0582	0.0285
Via CAD‐P time 1	0.0616		0.0578	0.0377		−0.0130	0.1355

The relationship between Time 1 and Time 2 ČEFSA scores was significantly mediated by the ES subscale of the ERQ‐CA (unstandardised indirect effect size = 0.0226, bootstrapped SE = 0.0105, bootstrapped 95% CI = 0.0033–0.0450). Higher Time 1 ČEFSA scores were associated with more use of ES (*b* = 0.0362, SE = 0.005, *p* < 0.001), which in turn was associated with higher Time 2 ČEFSA scores (*b* = 0.626, SE = 0.263, *p* = 0.018). A standardised indirect effect size of 0.212 indicates that for every standard deviation increase in ČEFSA score at Time 1, the Time 2 ČEFSA scores will increase by 0.212 standard deviations through ERQ‐CA (ES).

Neither the cognitive reappraisal subscale of the ERQ‐CA (unstandardised indirect effect size = 0.0058, bootstrapped SE = 0.0061, CI = −0.0055–0.0189), AQC scores (unstandardised indirect effect size = −0.0138, bootstrapped SE = 0.0220, CI = −0.0582–0.0285), nor CAD‐P scores (unstandardised indirect effect size = 0.0616, bootstrapped SE = 0.0377, CI = −0.0130–0.1355) were significant mediators.

## DISCUSSION

In this study, we have demonstrated using a large group of adolescents aged 13–18 years, that DE in young people are common. The majority (91.87%) reported experiencing at least one DE ‘often’ or ‘always’ over the past 2 weeks, and nearly a third (32.1%) experienced half or more of the 35 DE described by the ČEFSA scale. This corroborates previous findings that adolescents typically experience high rates of DE (Goffinet & Beine, 2018; Michal et al., 2015; Vine et al., 2020).

A novel finding from the current study, however, is that when surveying a broad range of common DE (Černis, Beierl, et al., 2021), for young people, such experiences most frequently take the form of detachment from the external world and other people (i.e., derealisation). Detachment from oneself in the form of anomaly in the experience of one's voluntary action was also highly endorsed, suggesting depersonalisation is also common in this group, and highlighting again the importance of the theme of control in DE (Ciaunica et al., 2024; Černis, Bird, et al., 2021; Černis et al., 2025). Experiences of unfamiliarity (e.g., *jamais vu*) were least common.

In terms of the psychological factors potentially explaining DE, this study finds some support for the theoretical model put forward by Černis and colleagues (Černis, Ehlers, & Freeman, 2022; Černis et al., 2025). The non‐significant result for alexithymia in this group supports its omission from the theoretical model, despite acknowledgements during model development that alexithymia may be an important factor consider in the context of FSA‐dissociation (Černis, Ehlers, & Freeman, 2022; Černis et al., 2025; Černis, Molodynski, et al., 2022). The significant result for ES and non‐significant result for cognitive reappraisal can also be interpreted as supporting the central premise of the model; that an aversion to affect plays a key role in dissociation (Černis, Ehlers, & Freeman, 2022; Černis et al., 2025). Cognitive reappraisal showed a negative relationship to DE, but was not a significant mediator, suggesting that countering negative interpretations of affect goes some way to reducing DE, but is insufficient (alone) to produce significant relief. This may imply that there is something uniquely important about the affective or embodied experience that a purely cognitive strategy cannot fully address—this is a hypothesis that merits further exploration. The significant result for ES is less ambiguous in its support of the theoretical model, and corroborates similar findings in adulthood and late adolescence. For example, Özgönül et al. (2023) demonstrate that reduced expression of emotions is associated with increased dissociation in 18–24 year olds, and Ó Laoide et al. (2018) found that clinically significant levels of dissociation (depersonalisation) in adults is predicted by negative attitudes toward emotion. In our study, the maladaptive emotion regulation strategy of ES (i.e., denying or avoiding affect) showed small but significant mediation of dissociation scores between timepoints. Given the relatively short time between these measurements (1 month), this represents a promising indication that the proposed theoretical model may be useful for working with younger clients.

Such a finding is intriguing from a neurocognitive perspective, and suggests new avenues for neuroimaging research. The prefrontal cortex is typically considered to continue maturing into a person's twenties (Starcevic & Filipovic, 2018) and is also implicated in the over‐regulation of the amygdala in the dissociative subtype of PTSD (Lanius et al., 2010). This raises questions about whether the prefrontal cortex plays the same role in adolescent dissociation as it does in adult dissociation (as modelled by dissociative PTSD), and how consciously accessible the self‐reported down‐regulation demonstrated in the current study relates to neurological development and any involuntary or automatic avoidance.

The aforementioned short duration between timepoints may explain the surprising non‐significant result for catastrophic cognitive appraisals, which has typically shown large and significant effect sizes in previous research (e.g., Černis, Bird, et al., 2021; Černis, Ehlers, & Freeman, 2022; Černis, Molodynski, et al., 2022), and follows the core premise of the cognitive‐behavioural model (Beck, 1979). One possible explanation may be that the relationship between cognitive appraisals and DE has the most impact within shorter timeframes (i.e., cognitive appraisals driving further DE during a ‘live’ experience of dissociation), with other factors holding more responsibility for the longer‐term persistence of DE once the initial response has passed. This would be in‐keeping with the conceptualisation of ‘negative automatic thoughts’ (Beck, 1979), as well as the model proposed by Černis and colleagues (Černis et al., 2025), which distinguishes between ‘foreground’ (proximal: classical CBT feedback loop) and ‘background’ (distal: affect intolerance and self‐efficacy feedback loop) processes. Another explanation for the absence of further mediation effects may be a high correlation between Time One and Time Two dissociation scores (*r* = 0.881 in the analysis by Shipp et al., 2023). Further research aiming to understand the maintenance factors of dissociation should therefore consider the use of EMA over the course of longer timeframes, and with more time between assessment points.

Alternatively, since any causal property of the relationships remains unclear in the current study due to its observational nature, experimental approaches may be preferable. For example, intervening on ES to test for subsequent improvement in dissociation scores. Indeed, Vancappel et al. (2025) recently showed that group CBT incorporating acceptance of emotions and identification of (mal)adaptive emotion regulation strategies improved dissociation in 27 adults with dissociative PTSD. However, it should be noted that the protocol also included numerous other helpful interventions, such as psychoeducation and cognitive restructuring, making it unclear how much of the effect on dissociation was due to alterations in affect avoidance. Nevertheless, such findings—alongside those presented here—indicate that maladaptive responses to affect may be an important treatment target for dissociation arising in adolescence and thus warrant further exploration in future treatment development work in this area.

Limitations of this study include the generalisability of the findings given the unrepresentative nature of the participant group, which was a majority White (84.69%) and female (66.61%) sample. It is also important to note that these data cannot indicate whether respondents were distressed by their experiences, or the level of any impact these may have had on activities of daily living. It is entirely possible, therefore, that DE are common in adolescence—but that levels of distress and impairment are not significantly higher. These data also cannot answer the question of absolute prevalence rates. Further investigation is required to fully understand these aspects, and caution is required in clinical settings—distress should be assessed, rather than assumed, where clients score highly on self‐report measures.

Finally, the current study does not consider additional dissociation measures, which precludes examination of the discriminant validity of FSA‐dissociation relative to other similar constructs (namely, depersonalisation or anomalous self‐experiences). Establishing such distinctions is an important goal for future research.

## CONCLUSION

This study found that DE are common in adolescents and most often take the form of depersonalisation and derealisation—particularly detachment from the external world, other people, and their own sense of agency. Despite highly correlated dissociation scores between close timepoints, our results still found that suppression of emotional expression was a significant mediator of dissociation over 1 month. This finding opens avenues for applying theoretical models of adult dissociation based on affect intolerance to younger populations, and raises new questions for neuroimaging research.

## AUTHOR CONTRIBUTIONS

**Emma Černis**: Conceptualization; writing—original draft; writing—review and editing; methodology; visualization; formal analysis; project administration; data curation; supervision. **Milan Antonović**: Writing—review and editing; formal analysis; data curation. **Katie Lofthouse**: Investigation; funding acquisition; writing—review and editing; data curation; project administration. **Lottie Shipp**: Investigation; writing—review and editing; project administration; data curation. **Polly Waite**: Conceptualization; writing—review and editing; methodology; supervision.

## CONFLICT OF INTEREST STATEMENT

The authors declare no conflicts of interest.

## ETHICAL CONSIDERATIONS

Informed consent/assent to participate was obtained from all participants, including parental consent where relevant. The studies contributing data to this study received ethical approval from the University of Oxford Medical Sciences Interdivisional Research Ethics Committee: R71497/RE001 (20^th^ October 2020) and R77368/RE001 (12th October 2021).

## Data Availability

The data that support the findings of this study are available on request from the corresponding author. The data are not publicly available due to privacy or ethical restrictions.
